# Characterizing the Gait of People With Different Types of Amputation and Prosthetic Components Through Multimodal Measurements: A Methodological Perspective

**DOI:** 10.3389/fresc.2022.804746

**Published:** 2022-03-17

**Authors:** Cristiano De Marchis, Simone Ranaldi, Tiwana Varrecchia, Mariano Serrao, Stefano Filippo Castiglia, Antonella Tatarelli, Alberto Ranavolo, Francesco Draicchio, Francesco Lacquaniti, Silvia Conforto

**Affiliations:** ^1^Department of Industrial, Electronics and Mechanical Engineering, Roma Tre University, Rome, Italy; ^2^Department of Engineering, University of Messina, Messina, Italy; ^3^Department of Medicine, Epidemiology, Occupational and Environmental Hygiene, National Institute for Insurance Against Accidents at Work (INAIL), Rome, Italy; ^4^Department of Medical-Surgical Sciences and Biotechnologies, Sapienza University of Rome, Latina, Italy; ^5^Department of Human Neurosciences, Faculty of Medicine and Dentistry, Sapienza University of Rome, Rome, Italy; ^6^Department of Systems Medicine and Center of Space Biomedicine, University of Rome Tor Vergata, Rome, Italy

**Keywords:** gait analysis, neuromechanics, prostheses, multimodal characterization, electromyograhy (EMG), muscle synergies, lower limb amputation

## Abstract

Prosthetic gait implies the use of compensatory motor strategies, including alterations in gait biomechanics and adaptations in the neural control mechanisms adopted by the central nervous system. Despite the constant technological advancements in prostheses design that led to a reduction in compensatory movements and an increased acceptance by the users, a deep comprehension of the numerous factors that influence prosthetic gait is still needed. The quantitative prosthetic gait analysis is an essential step in the development of new and ergonomic devices and to optimize the rehabilitation therapies. Nevertheless, the assessment of prosthetic gait is still carried out by a heterogeneous variety of methodologies, and this limits the comparison of results from different studies, complicating the definition of shared and well-accepted guidelines among clinicians, therapists, physicians, and engineers. This perspective article starts from the results of a project funded by the Italian Worker's Compensation Authority (INAIL) that led to the generation of an extended dataset of measurements involving kinematic, kinetic, and electrophysiological recordings in subjects with different types of amputation and prosthetic components. By encompassing different studies published along the project activities, we discuss the specific information that can be extracted by different kinds of measurements, and we here provide a methodological perspective related to multimodal prosthetic gait assessment, highlighting how, for designing improved prostheses and more effective therapies for patients, it is of critical importance to analyze movement neural control and its mechanical actuation as a whole, without limiting the focus to one specific aspect.

## Introduction

The amputation of a lower limb is a complex and invasive surgery that is often needed due to traumatic events, vascular diseases, or tumors. The changes in demographics and the increasing incidence of the pathologies leading to an amputation will potentially impact the healthcare services, including the demand of prosthetic devices, and the number of persons living with the loss of a limb has been estimated to significantly increase in the next few decades (1). People with a lower limb amputation reported a quality of life (QoL) that is significantly lower with respect to the general population. Among others, factors such as the use of a specific prosthesis and close related factors such as residual stump pain and type of suspension were found to predict QoL scores significantly (2–6).

After the surgery, people with amputation must undergo a rehabilitation phase and a considerable walking training (7) to gain the ability to walk autonomously and safely with a prosthetic device (8, 9). People with mono-lateral amputation typically adopt a series of compensatory motor strategies involving the prosthetic side and the contralateral sound limb (10, 11), plus an increased involvement of pelvis and trunk (12–16). As a matter of fact, prosthetic gait reflects a mixture of deviations from normal gait and adaptive and compensatory motions dictated by residual limb functions. From a motor control standpoint, during the rehabilitation process, people with amputation must adapt their walking patterns to their new physical conditions, and this adaptation may result in changes in the way the central nervous system (CNS) controls the movement. Lower limb amputation leads to significant neural reorganization within the CNS, mostly due to the loss of the sensorimotor function caused by amputation (17) and to the new biomechanical condition induced by the type of amputation and by the used prosthetic device. The two factors influencing the gait in people with amputation are the level of the amputation (18, 19) and the type of prostheses (20–27). Regarding the former factor, the gait in people with transfemoral amputation (TFA) seems to be more asymmetric than that in people with transtibial amputation (TTA), with increased compensatory strategies, which, over time, may prove damaging to individuals (28). Concerning the latter factor, in recent years, the prostheses have improved in design, materials, and technology (29–32) to be more effective in terms of efficiency of ambulation, minimization of the asymmetries, and reduction of compensatory movements.

In this scenario, quantifying and characterizing the gait of persons with a prosthesis is an essential element to improve the development of new and ergonomic prosthetic devices, and to optimize the rehabilitation programs (33–36). Nevertheless, the heterogeneous variety of methodologies used to assess prosthetic gait limits the comparison of results from different studies and complicates the definition of shared guidelines. The quantitative prosthetic gait assessment should be focused on indicators of effective and ecological gait, such as the traditional gait parameters, level of gait asymmetries, metabolic consumption, and the amount of compensatory muscle activation, but the assessment of such an heterogeneous scenario requires novel research methodologies (37–41).

From this standpoint, the adoption of a multimodal approach is needed for a proper prosthetic gait evaluation. The project “Modular motor control of the contralateral sound limb in people with lower limb amputation: neuromechanical assessment of the prosthetic components in the control of locomotion,” funded by the Italian Worker's Compensation Authority (INAIL), led to the generation of an extended dataset comprising multimodal measurements involving all the common gait analysis instruments. By discussing the results of different studies published within the project, identifying some peculiarities in the used instrumentation and highlighting the importance of the related indices, we here provide a methodological perspective related to multimodal prosthetic gait assessment.

Starting from the dataset recorded during the project activities in the first section, we then describe the results obtained in five different studies, published along the project activities that analyze specific aspects of gait of people with amputation. Each study yields both direct evidence, coming from the recorded data and based on the specific indices used to quantify the gait performance, and indirect evidence emerging from the interpretation of the results. These direct evidence and indirect interpretations are then summed up and integrated in the final perspective section of the article, where we provide a methodological perspective supporting the importance of multimodal prosthetic gait assessment.

## Population, Measurements, and Protocol

The population enrolled during the project activities underwent a typical gait analysis protocol executed with a multimodal set of measurements in terms of instrumentation and variable number of subjects, as reported in Table 1. This perspective article takes into account the results of 5 different studies. All the experiments were carried out at the Rome Branch of Prosthetics Center of INAIL, at the CTO Andrea Alesini hospital of Rome.

**Table 1:** Populations characteristics across the 5 studies.

	**Without EMG**			**With EMG**	
**Population age height mass**	**Varrecchia et al. (42)**	**Castiglia et al. (43)**	**Ranaldi et al. (44)**	**De Marchis et al. (45)**	**Tatarelli et al. (46)**
TFA_M_	*n =* 9 (1 F) 56.9 ± 12.6 yo 169.5 ± 4.9 cm 79.8 ± 16.8 kg	*n =* 9 (1 F) 56.9 ± 12.6 yo 169.5 ± 4.9 cm 79.8 ± 16.8 kg			*n =* 10 (1 F) 58.7 ± 11.5 yo 171.3 ± 7.0 cm 79.7 ± 15.9 kg
TFA_C_	*n =* 17 (2 F) 58.2 ± 14.0 yo 172.3 ± 7.4 cm 83.0 ± 13.1 kg	*n =* 17 (2 F) 58.2 ± 14.0 yo 172.3 ± 7.4 cm 83.0 ± 13.1 kg	*n =* 7 54.6 ± 14.6 yo 173.4 ± 6.4 cm 84.4 ± 16.9 kg	*n =* 7 54.6 ± 14.6 yo 173.4 ± 6.4 cm 84.4 ± 16.9 kg	*n =* 16 (2 F) 56.4 ± 14.1 yo 172.4 ± 6.9 cm 81.7 ± 13.5 kg
TFA_G_	*n =* 14 50.2 ± 12.8 yo 177.5 ± 16.0 cm 88.0 ± 13.0 kg	*n =* 14 50.2 ± 12.8 yo 177.5 ± 16.0 cm 88.0 ± 13.0 kg	*n =* 7 46.8 ± 14.5 yo 177.0 ± 7.2 cm 87.0 ± 13.8 kg	*n =* 7 46.8 ± 14.5 yo 177.0 ± 7.2 cm 87.0 ± 13.8 kg	*n =* 11 48.4 ± 13.5 yo 177.5 ± 5.8 cm 84.6 ± 12.2 kg
TTA	*n =* 15 52.8 ± 14.5 yo 176.4 ± 5.4 cm 87.4 ± 11.1 kg				*n =* 11 59.4 ± 12.8 yo 176.0 ± 6.2 cm 85.1 ± 12.7 kg
C	*n =* 40 (3 F) 54.9 ± 12.3 yo 172.8 ± 7.9 cm 83.5 ± 15.7 kg		*n =* 12 53.6 ± 8.1 yo 176.9 ± 7.0 cm 78.2 ± 6.6 kg	*n =* 12 53.6 ± 8.1 yo 176.9 ± 7.0 cm 78.2 ± 6.6 kg	*n =* 22 (3 F) 52.8 ± 14.5 yo 176.4 ± 5.4 cm 87.4 ± 11.1 kg

In total, 57 recordings from subjects with unilateral TFA and 20 recordings from subjects with unilateral TTA were performed. The subjects with TFA wore three different types of prostheses: mechanical prosthesis (TFA_M_) and two types of prostheses with microprocessor-controlled knees (MPKs), namely C-Leg (TFA_C_) and Genium (TFA_G_) prosthesis (Ottobock, Duderstadt, Germany). All subjects with TFA and TTA were provided with the same type of prosthetic foot (Ossur Variflex, Reykjavik, Iceland), whereas the socket was custom-made and adapted to the single user needs before the gait analysis protocol by an experienced physician. All subjects with lower limb amputation were experienced prosthesis users (i.e., able to walk safely with a prosthetic device for more than 2 years). In addition to the TFA and TTA populations, 40 healthy subjects were recruited as the control group (C), and they were age–sex–speed matched with the amputees group.

Walking tests at a self-selected comfortable speed were performed on a 9-m long walkway instrumented with two force platforms (Kistler9286AA, Winterthur, Switzerland). Control subjects were requested to walk also at a lower speed to match the TFA and TTA groups. A six-infrared camera optoelectronic motion analysis system (SMART-DX 6000 System, BTS, Milan, Italy) was used, with passive spherical markers placed according to a modified Davis' protocol (47). In subjects with TTA and TFA, the amputated limb markers were placed over symmetrical points with respect to the homologous marker's position on the non-amputated limb. Electromyographic (EMG) signals were recorded using a wireless system (FreeEMG 1000 System, BTS, Milan, Italy). Muscle activity was recorded from 12 muscles of the sound side (right side for the controls).

## Results

The results coming from the multimodal analysis underlying this paper are briefly reported in Table 2 in terms of their direct and indirect interpretations. The following paragraphs report details on the mentioned studies that will serve as a base for the final perspective about the emergent features of prosthetic gait that are shown by adopting this kind of analytical approach.

**Table 2:** Direct and indirect evidence and interpretations emerging from the findings of 5 different studies.

	**A-Spatiotemporal**		**B-Kinematic/Kinetic**		**C-Energy consumption**		**D-Motor control**	
	**Direct**	**Indirect**	**Direct**	**Indirect**	**Direct**	**Indirect**	**Direct**	**Indirect**
I-Varrecchia et al. (42)	Some patterns are typical of TFA gait	More advanced prosthetic knees perform better in gait	Compensatory mechanisms happen through pelvis and trunk	Compensation of lack of sensory feedback and foot placement TFA unable to generate adequate forces during stance		Less advanced prosthetic knees need higher compensatory effort		
II-Castiglia et al. (43)			Normalization of pelvic obliquity on the prosthetic side of the subjects using more advanced prosthetic knees	Pelvic obliquity is related to “hip hiking” strategy of the affected side Pelvic obliquity allows limb forward progression during walking	Pelvic obliquity affects energy recovery	More advanced prosthetic knees can reduce the risk of low back pain		
III-Tatarelli et al. (46)				Increased coactivation reflects the compensatory increase in stiffness and changes in force production capacity Compensatory coactivation of the sound limb muscles may relevantly contribute to asymmetry		Compensatory coactivation of the sound limb muscles may relevantly contribute to excessive energy expenditure		The most critical phases in prosthetic gait are the double support ones Differences in coactivation depend more on the inertial properties of the prostheses rather than control mechanisms
IV- De Marchis et al. (45)				Same synergies between TFA and controls indicate same biomechanical functions		Synergy activation modifications during weight transfers represent an efficient compensatory mechanism	Motor coordination schemes in TFA are not different from the case of non-pathological gait	The most critical phase in TFA gait is the weight transfer phase from the sound limb to the prosthetic one. Alterations in synergy recruitment constitute a speed independent marker of TFA gait
V-Ranaldi et al. (44)		Principal components of elevation angles might be related with the spatiotemporal gait parameters						Double support phases are the most critical to be managed in prosthetic gait Alterations in principal components characteristics are related to altered neuromuscular control strategy

### Kinematic, Kinetic, and Energy Consumption Patterns

By including two different amputation levels (i.e., TFA and TTA) and three different types of prostheses for the TFA (i.e., mechanical, C-Leg, and Genium), a comparison of spatiotemporal parameters, plus kinematic and kinetic indicators, as compared to a speed-matched control group, was conducted in Varrecchia et al. (42).

The study highlighted that some patterns characterize prosthetic gait in general, regardless of the type of amputation and the kind of used prosthesis, whereas the others are specific for TFA and are dependent on the type of prosthetic knee (I-A_DIR_).

From a purely kinematic standpoint, TFA and TTA subjects show an increased step width, step length, and double support duration. These subjects also show an increased pelvic obliquity and a higher range of motion (RoM) in trunk movements when compared with controls, regardless of whether the leading limb was the prosthetic one or the sound one, indicating that most of the compensation happens through the pelvis and trunk (I-B_DIR_). An increased stance/swing ratio characterizes the sound side. From a kinetic standpoint, the prosthetic gait is characterized by an increased initial peak in the ground reaction force (GRF) on the sound side. All these alterations might be due to a lack of sensory feedback and to an absence of perception regarding foot placement (I-B_IND_).

However, besides these common alterations in gait patterns, some additional changes characterize the gait of people with TFA. Kinematic alterations include a reduced stance/swing ratio in the prosthetic side and a higher hip and knee RoM in the sound side. Kinetic alterations include an increased initial peak in the GRF on the prosthetic side, suggesting that TFA are not able to generate adequate forces during stance (I-B_IND_).

When considering the effect of the device, subjects using a Genium prosthesis have a lower pelvic obliquity when compared to TFA_M_, a higher hip and knee RoM on the prosthetic side and an increased step length when the sound limb leads. This might indicate that more advanced knee prostheses have a general better performance in gait (I-A_IND_), leading to a reduced compensatory effort (I-C_IND_).

Since the main alterations are present in the TFA gait, the potentially induced increase in the metabolic consumption could be explained by an energy-related indicator able to discriminate among different types of prostheses, shedding light onto the efficacy of different prosthetic components. Compared with speed-matched healthy controls, subjects with TFA, indeed, are characterized by a lower ability to recover mechanical energy at each walking step, regardless of the type of prostheses. Among the various spatiotemporal and kinematic modifications in the TFA gait, the only variable that is related to the lower energy recovery is the pelvic obliquity on the prosthetic side (43) (II-C_DIR_). The pelvic obliquity is the rotation of the pelvis around the coronal plane, defined as the angle between the horizontal plane and the mediolateral axis of the pelvis, and its increase has been shown to be a compensation strategy used to propel the limb and recover energy (II-B_IND_). This parameter not only correlates with relevant energy consumption measurements, but also highlighted that most advanced technological prostheses, such as Genium, likely require less compensation in the pelvic obliquity to recover the same amount of energy at each walking step (II-B_DIR_), thus potentially reducing the risk of low back pain (II-C_IND_).

### Electrophysiological Features of Prosthetic Gait

Although these considerations allow us to better understand the effect of prosthetic gait on the movement mechanical outcome, yet the causes leading to such modifications can only be estimated, and the underlying changes in the control strategies adopted by the neuromuscular system can be roughly inferred but cannot be quantitatively accessed. A multimuscle activity measurement involving the sound limb has been performed to further advance our comprehension on the underlying neuromuscular strategies, by recording the EMG activity of 12 mono- and bi-articular muscles acting at the ankle, knee, and hip joints.

To better understand the coordination mechanisms of such muscles, a compact indicator, consisting of a time-varying function, has been used to describe the global neuromuscular strategy adopted by a subject in modulating the simultaneous activation/deactivation of many muscles during gait (48). The analysis on a population of TTA, TFA, and controls highlighted that people with amputation had a coactivation profile similar to the control population. However, the prosthetic gait led to an increased level of simultaneous activation during the loading response and push-off phases, whereas this coactivation was decreased during midstance and swing (46). This increased coactivation probably plays a role in the prosthetic gait asymmetry and altered energy consumption (III-C_IND_). Among people with TFA, the used kind of prosthesis had an effect on the global coactivation, as it resulted lower in C-Leg users when compared with Genium and mechanical prostheses users. In detail, the increased coactivation levels that are recorded during the general prosthetic gait can be seen as a cause for the decreased force generation capacity and as an additional source of asymmetry (III-B_IND_). Moreover, the same coactivation can also be seen as an important feature of motor control, isolating the double support phases of gait as the most critical for walking with a prosthesis, in which the different inertia properties of a prosthetic leg with respect to the intact limb might play a key role (III-D_IND_).

### Neuromechanics and Motor Control

The aforementioned multi-muscle EMG recording can also take advantage of the nowadays widespread and clinically relevant theory of modularity in motor control (49). By using the synchronous muscle synergy model, it was possible to identify low-dimensional control structures characterizing the muscle coordination of TFA subjects. In De Marchis et al. (45), it was shown that, despite the visible alterations in muscle activity, the complexity in muscle coordination did not change, as the TFA group exhibited 4 modules, which is the same number of muscle synergies typically expressed by control populations (IV-D_DIR_). When analyzing the spatial structure of these modules (i.e., the groups of muscles working synergistically), it was shown that it is shared between TFA and controls, consisting of a weight acceptance module at sound limb heel strike, a propulsion module before toe-off, a swing module, and a late swing deceleration module before heel strike. This indicates that the main underlying biomechanical functions were preserved (IV-B_IND_). However, the difference between TFA and controls was clearly visible in the activation of two out of the four identified modules: a significantly prolonged activation of the propulsion module (calf muscles) and an abnormal activation of the late swing deceleration module (hamstring muscles) during the second double support phase with respect to speed-matched controls (IV-D_IND_). This result indicates that the most critical phase in gait of people with TFA is the second double support phase, corresponding to the weight transfer from the sound limb to the prosthetic one (IV-D_IND_), potentially reflecting an efficient compensatory mechanism that enforces the interpretation of the results on the coactivation strategies (IV-C_IND_).

Analogous results can be obtained by exploiting the planar covariation law of elevation angles. Following the same rationale adopted for the muscle synergy analysis, it is possible to define a common spatial organization for the behavior of the elevation angles of the three lower limb segments (50). With this description, both limbs of the patients and healthy subjects share the same covariation domain, with differences that are limited to the trajectories of the three angles in this space (44) (V-D_IND_). Coherently with all the other studies presented before, most of the differences are to be ascribed to the management of the body weight and on the contact phase of the limb with the ground (i.e., the stance phase), with the prosthetic limb showing a higher degree of correlation among the three leg segments, as a direct consequence of the control mechanisms of the prosthetic knee (V-D_IND_). Moreover, the planar covariation law of elevation angles is a compact description that directly approximates the kinematics of the two legs; as a consequence, it is ideally possible to exploit this economic description of gait for predicting different quantitative measures of walking behavior, such as the spatiotemporal parameters, giving rise to a variety of applications for prosthetic control and rehabilitation (V-A_IND_).

## Discussion

Previous studies have highlighted some important features of prosthetic gait, including stability, spatiotemporal gait parameters, and symmetry, gathering relevant information by using a reduced set of sensors (51–57). However, due to the heterogeneous nature of the prosthetic components, type of rehabilitation, experience with prosthesis use, and the amputation characteristics themselves, a multimodal approach to such gait analysis could be able to shed light on some important features related to these multiple factors (38, 58, 59), thus supporting the clinical practice (60–62). The neuromechanical analysis of gait, bringing together the information on biomechanical aspects and neural control aspects, takes advantage of this multimodal approach. This adds to the lack of studies including an EMG analysis of the contralateral limb, which provides a powerful insight into how the CNS is adapting to walking with a prosthesis (63–65), in addition to the changes appearing at the level of the residual musculature (66, 67). Overall, kinematic, kinetic, and surface EMG gait findings reflect the compensatory efforts developed by people with amputation to protect the soft tissues of the prosthetic limb and to deal with the new prosthetic limb condition.

Within the framework of the INAIL-funded project activities, the aim of this perspective is to fill the gap between the complexity of prosthetic gait and the necessity of a complete set of measurements. The summary of the discussions of the outcomes of the project presented here, reported in Table 2, highlights how different analyses can yield a wide overview of the characteristics of the prosthetic gait, reducing the number of indirect considerations (i.e., speculations) that are needed to describe all the factors starting from the results of an incomplete set of analyses. Table 2 reports, for each of the five studies, both the direct evidence, as obtained from the analysis of specific quantities of prosthetic gait, and the indirect interpretations related to aspects that are not explicitly considered in the analysis. These are discussed in the following sections.

### Spatiotemporal, Kinematic, Kinetic Metabolic, and Motor Control Aspects of Prosthetic Gait

From a spatiotemporal parameters point of view, a typical gait analysis can be combined with the analysis of the coordination of the lower limb segments, linking concepts related to the biomechanics to motor control investigations. Although the analysis of the spatiotemporal parameters of gait is well-established in the scientific literature, it leads to results that are not directly linked to how the movement is controlled, and thus might fail in identifying the key adaptation mechanisms that underlie walking with a prosthesis and the variables relevant to patient's satisfaction (68). In this scenario, the methodological approaches from I (42) and V (44) could take a strong advantage from a joint analysis, with important implications in engineering (e.g., development of advanced control systems for prostheses based on the underlying motor control mechanisms) and clinical practice (e.g., the use of motor control theories as a benchmark for functional gait recovery).

When dealing with kinematic and kinetic analysis of gait, the multimodal approach is of critical importance for a correct interpretation of the results. This happens with the interpretation of the results of both standard gait and coactivation analyses, from which it is possible to only indirectly suppose that prosthetic gait is characterized by a lower capacity of force generation, whereas the combination of the two studies reinforces this hypothesis. These considerations suggest that the methodologies of I (42) and III (46) have an important complementary role in the assessment of prosthetic gait dynamics.

Moreover, the synergy analysis confirms that the same biomechanical functions of physiological gait are preserved but controlled with different timings; by combining these results with those on pelvic obliquity and general kinematics, it is possible to understand how the abnormal activations in the synergy profiles reflect on the altered movement biomechanics. In the same manner, both the discussions about motor control and energy consumption can be summarized by focusing on the difficulties and asymmetries in the management of the weight shift phases (at the beginning and the end of the stance phase of both legs); this, combined with the results on the pelvic obliquity and with the characterization of the energetic inefficiency, confirms in a quantitative way the already published results, that identify the double support phases of prosthetic gait as the most critical, both from a stability and an energetic point of view. These considerations highlight the importance of connecting the multi-muscle EMG measurement and synergy analysis of IV (45) with the methodologies used in II (43) related to the body center of mass and to pelvic kinematics, for a complete understanding of the interplay between compensation mechanisms and energy consumption.

### Perspective of the Multimodal Analysis of Prosthetic Gait

Future studies should explore whether the adoption of a multimodal approach can capture the alterations in performance-based walking measures (69), the metabolic cost of walking (32, 70), self-perceived mobility and balance outcomes (37, 71), and the acceptance of prostheses (3). In the clinical practice, the outcomes of the rehabilitation therapies are often measured by means of qualitative scales, such as the K-Level, which to date is the main scale used by physicians to choose the most adequate prosthetic device. Some semi-quantitative scales have also been proposed, like the Amputee Mobility Predictor (AMP) scale (72), in which some spatiotemporal parameters of gait are used to refine the information provided by the K-level classification. In addition to these indices, several clinical scales describing the patient's QoL are adopted as a description of the follow-up of the therapies, such as the Amputee Activity Survey or the 12-min walking test (73). In general, all the current clinical scales are pseudo-subjective, based on questionnaires that are dependent on the personal perception of either the physician or the patient. Nevertheless, they might fail in providing insight into the interplay between the prosthesis and the patient so that the adopted solutions might not be always optimal. Although it remains still feasible to use those measures in the clinical practice, the recent advancement in prosthesis technology could make the patients reach high scores for most of these scales. In this sense, the definition of more objective scales, exploiting engineering tools, can help in reaching a higher level of understanding of how the choices of the prosthetic component affect the movement control; this can lead to the development of prosthetic devices that reach a higher degree of integration with the subject's motor control strategies.

## Conclusions

The meta-discussion presented here was elicited by the heterogeneous framework of results obtained within the project “Modular motor control of the contralateral sound limb in people with lower limb amputation: neuromechanical assessment of the prosthetic components in the control of locomotion” funded by INAIL. All the considerations from the related studies strongly highlight the importance of applying a multimodal approach when analyzing gait in people with a lower limb amputation; as a matter of fact, despite the huge scientific effort of the last two decades, this condition is still partially unknown to date, and the compensations that are necessary for reaching stable gait with a prosthesis are highly complex and cannot be characterized as a whole without a complete recording and analysis of all the influence factors. Consequently, this strongly recommends that, for designing improved prosthetic device, develop more advanced and physiologically inspired prosthesis control systems, and plan more effective therapies for these patients, it is of critical importance to analyze movement neural control and mechanical actuation as a whole, without limiting the focus to one specific aspect.

## Data Availability Statement

The original contributions presented in the study are included in the article/supplementary material, further inquiries can be directed to the corresponding author.

## Ethics Statement

Ethical approval was not provided for this study on human participants because it analyses previously published results. Details on ethics can be found in the original referenced articles. The patients/participants provided their written informed consent to participate in this study.

## Author Contributions

CD, SR, and SFC conceptualized the study. CD and SR prepared the original draft of the manuscript. SC, AR, MS, FD, and FL provided resources and administered the project. All authors reviewed and edited the manuscript for important intellectual content.

## Funding

This research was funded by INAIL, Bando Ricerche in Collaborazione 2016.

## Conflict of Interest

The authors declare that the research was conducted in the absence of any commercial or financial relationships that could be construed as a potential conflict of interest.

## Publisher's Note

All claims expressed in this article are solely those of the authors and do not necessarily represent those of their affiliated organizations, or those of the publisher, the editors and the reviewers. Any product that may be evaluated in this article, or claim that may be made by its manufacturer, is not guaranteed or endorsed by the publisher.
